# Anti-tumor Synergistic Effect of a Dual Cancer-Specific Recombinant Adenovirus and Paclitaxel on Breast Cancer

**DOI:** 10.3389/fonc.2020.00244

**Published:** 2020-03-25

**Authors:** Jing Wang, Yiquan Li, Shanzhi Li, Wei Yao, Xing Liu, Yilong Zhu, Wenjie Li, Liankun Sun, Ningyi Jin, Xiao Li

**Affiliations:** ^1^Department of Pathophysiology, College of Basic Medical Sciences, Jilin University, Changchun, China; ^2^Institute of Military Veterinary Medicine, Academy of Military Medical Science, Changchun, China; ^3^Academician Workstation of Jilin Province, Changchun University of Chinese Medicine, Changchun, China; ^4^Center for Disease Control and Prevention, Agency for Offices Administration, Central Military Commission, Beijing, China; ^5^Department of Thoracic Surgery, The First Hospital of Jilin University, Changchun, China; ^6^Jiangsu Co-innovation Center for Prevention and Control of Important Animal Infectious Diseases and Zoonoses, Yangzhou, China

**Keywords:** recombinant adenovirus, paclitaxel, toxicity, synergy, breast cancer

## Abstract

This study aimed at investigating the anticancer potential of the recombinant adenovirus Ad-apoptin-hTERTp-E1a (Ad-VT) and its synergistic combination with paclitaxel (PTX) in breast cancer treatment. First, we used the Calcusyn software to analyze the synergy between the Ad-VT and paclitaxel, and to determine the final drug concentration. Second, we used crystal violet staining and WST-1 assays to analyze the inhibitory effect of Ad-VT and paclitaxel combination treatment on MCF-7, MDA-MB-231, and MCF-10A cells. Subsequently, we used Hoechst, Annexin V, JC-1 staining to analyze the inhibition pathway of drugs on breast cancer cells. We also used Transwell assays to analyze the cell migration and invasion of MCF-7 and MDA-MB-231 cells. The pGL4.51 plasmid was used to transfect and to generate MDA-MB-231 cells, that stably express luciferase (MDA-MB-231-LUC). The *in vivo* tumor inhibition effect of Ad-VT and paclitaxel combination treatment was subsequently confirmed using a tumor-bearing nude mouse model. This combination treatment can increase the inhibition of breast cancer cells and reduce paclitaxel toxicity. Ad-VT had a strong apoptosis-inducing effect on MCF-7 and MDA-MB-231 cells, that was mainly mediated through the mitochondrial apoptotic pathway. The combination of Ad-VT and paclitaxel could significantly increase the inhibition of breast cancer cell migration and invasion. Combination of Ad-VT and paclitaxel can inhibit tumor growth and reduce toxicity *in vivo*. Ad-VT can also inhibit the growth of breast cancer cells and promote their apoptosis. Meanwhile, when it is combined with paclitaxel, Ad-VT could play a significant role in a synergistic tumor inhibition.

## Introduction

Cancer is one of the most important public health problems in the world. With the changing disease structure and the aging trend of the population, the global burden of cancer has become increasingly prominent. Breast cancer is the cancer with the highest incidence rate among women in the world (1), accounting for about 46.3% of the overall cancer incidence rate and 13.0% of the mortality rate. Thus, breast cancer is a serious threat to women's health and its risk factors have also been a hot topic. In recent years, research has found that the main risk factors are classified into the following categories: (1) Aging (the incidence of breast cancer is highly correlated with increasing age); (2) Reproductive factors (such as early menarche, late menopause, late age at first pregnancy and low parity); (3) Dietary factors (including alcohol consumption, intake of soy products, dietary habits, etc.); (4) drug effects (including oral contraceptives, non-steroidal anti-inflammatory drugs, exogenous estrogens, etc.) and (5) genetic factors (2).

Currently, the most common treatment for breast cancer is surgery combined with radiotherapy and chemotherapy (Such as taxanes, anthracyclines, and cyclophosphamide, gemcitabine, cisplatin, etc.). Although both methods are effective, they also have significant side effects, preventing patients from obtaining high-quality life assurance. With developments in molecular biology, cell biology and virology, gene therapy has become an emerging mean of cancer treatment, in which Oncolytic virotherapy has shown great advantages, and is also expected to be a reliable way to treat breast cancer.

Oncolytic viruses (OVs) are emerging as important agents in cancer treatment as they offer the attractive therapeutic combination of tumor-selective cell lysis and by acting as potential *in situ* tumor vaccines (3–8). Early clinical trials of OVs showed encouraging safety profiles, even at high doses, and with some promising responses, such as the evidence of intratumor viral replication and efficient killing of tumors (9–11).

*In vivo* imaging tools will help to understand the molecular mechanisms that lead to cancer progression, metastasis, and chemo resistance. It is very important to develop preclinical research tools that ensure faster and more accurate analyses of the molecular pathways, that are critical in improving diagnosis, the design and screening of new drugs, and cancer treatment. *In vivo* bioluminescence imaging is a visualization technique used to track cellular, tissue activity and genetic behavior *in vivo* (12, 13). In this study, we transfected cells with a plasmid containing the luciferase gene, screened them with selective antibiotics until a single resistant clone emerged, and finally the clones with the best luciferase activity and stability were selected. The labeled tumor cells were injected into the animal to establish a visualized tumor model.

Apoptin is a small apoptosis-inducing protein derived from the chicken anemia virus (CAV), which belongs to the genus Circoviridae and possesses a single-stranded minus-strand circular DNA (14–16). It is a small 14 kDa protein that is rich in proline, serine, threonine and basic amino acids. Apoptin has the ability to selectively kill various human tumors or transformed cells, with little cytotoxic effect on normal cells (17). It contains a two-core nuclear localization signal and a nuclear export signal that facilitates protein shuttle between the nucleus and the cytoplasm, and has several potential phosphorylation sites, including on threonine-108 (thrl-108). Apoptin is specifically phosphorylated on thr108 in tumor cells, and is not observed in normal cells (18–21). The tumor specific phosphorylation of Apoptin has generated interest in identifying cellular kinases with increased activity in tumor cells, and that might be responsible for Apoptin phosphorylation and its tumor-specific activation.

The length and viability of the human telomerase reverse transcriptase (hTERT) is related to cell senescence and immortalization. Telomerase is an RNA-dependent DNA polymerase that elongates 5′-TTAGGG-3′ telomeric DNA (22). Most normal human somatic cells lack telomerase activity due to the tight transcriptional suppression of the rate-limiting and catalytic component of the telomerase reverse transcriptase (*hTERT*) gene. However, hTERT expression and telomerase activation are observed in up to 90% of human malignances, giving them unlimited proliferation ability (23). Studies have shown that the targeting of tumor cells and efficient expression of proteins of interest, is also dependent on the high efficiency and specificity of the hTERT promoter. This opens up new potential avenues for tumor therapy (24, 25). In previous studies, we took advantage of the characteristics of Apoptin and the hTERT promoter to construct an Apoptin expressing tumor-specific replication recombinant adenovirus (Ad-Apoptin-hTERTp-E1a, Ad-VT) (26). This allowed the adenovirus to specifically replicate in large numbers, within tumor cells; therefore, expressing Apoptin and leading to Apoptin-mediated tumor cells death. Additionally, we have shown that the recombinant adenovirus has a significant killing effect on several other tumor cells (27–30).

In the present study, we analyzed the synergistic concentration between the oncolytic adenovirus Ad-VT and the chemotherapeutic drug paclitaxel. We also studied the inhibitory effect of Ad-VT and paclitaxel combined treatment of breast cancer cells, using various *in vitro* experiments and a BALB/c nude mouse subcutaneous tumor model. The findings of this study provide a theoretical basis for the treatment of breast cancer using oncolytic adenoviruses and chemotherapy as a combination therapy.

## Materials and Methods

### Cells, Viruses, and Animals

Cryopreserved human breast cancer cells MCF-7, MDA-MB-231, and human normal mammary epithelial cells MCF-10A were purchased from the Cell Bank of the Shanghai Institute for Biological Science (Shanghai, China), and maintained in RPMI 1640 medium supplemented with 10% fetal bovine serum (FBS), 50 U/mL penicillin and 50 U/mL streptomycin, and incubated at 37°C with 5% CO_2_. All cell culture reagents were purchased from HyClone GE Healthcare Life Sciences (Logan, UT, USA).

The recombinant adenoviruses Ad-Apoptin-hTERTp-E1a (Ad-VT) and Ad-MOCK were constructed and preserved in our laboratory (Laboratory of Molecular Virology and Immunology, Institute of Military Veterinary Medicine, Academy of Military Medical Science, Changchun, China) (26).

Female BALB/c nude mice aged 4–5 weeks were purchased from the Experimental Animal Center of the Academy of Military Medical Sciences of China. All animal experimental protocols were approved by the Institutional Animal Care and Use Committee (IACUC) of the Chinese Academy of Military Medical Science, Changchun, China (10ZDGG007). All surgeries were performed under anesthesia with sodium pentobarbital and with adequate invasive animal procedures.

### Determination of Cytotoxic Synergy

The inhibition ratio of MCF-7 and MDA-MB-231 cells were examined by the WST-1 assay at 72 h. We first examined the inhibition rate of MCF-7 cells that were treated with 10, 50, and 100 MOI (multiplicity of infection) of Ad-VT, and 2, 4, 6, 8, and 10 nmol of paclitaxel. Subsequently, the concentration range was narrowed, and the inhibition rate was measured in MDA-MB-231 cells. The final concentration to use was determined, and its synergistic effect was verified in MCF-7 cells. The combination index (CI) values were analyzed using the Calcusyn software, which calculates CI values using the following equation: CI = (D)1/(Dx)1 + (D)2/(Dx)2 + (D)1(D)2/(Dx)1(Dx)2, where (Dx)1 and (Dx)2 are the doses for x% inhibition by drug 1 and drug 2 alone. (D)1 and (D)2 are the combination doses that inhibit cell growth by x%. A CI value of 1 indicates additive effects of the two agents, while a CI value >1 indicates antagonism effects, and <1 indicates synergism effects (31).

### Detection of the Inhibition Rate in Breast Cancer Cells

Inhibition of breast cancer cells by viral and chemotherapeutic drugs was examined by the crystal violet staining assay and the WST-1 assay.

MCF-7, MDA-MB-231, and MCF-10A cells were prepared as cell suspensions, and added to 12-well plates at a concentration of 2 × 10^5^ cells/ml. For each well, 1 mL of cell suspension was added. After incubating for 24 h with a mixture of the Ad-VT MOI of 50 and 4 nmol of paclitaxel, 50 MOI of Ad-VT, 50 MOI of Ad-MOCK, and 4 nmol of paclitaxel mixture was added. Blank control wells were also set. After 72 h, the cells were removed and stained with crystal violet. The culture media in the wells was discarded, the cells were washed three times with PBS, and then stained with 0.4% of crystal violet stain for 5 min. Afterward, the dye was discarded, and the cells washed three times with PBS, and used for photography.

The MCF-7, MDA-MB-231, and MCF-10A cells were prepared as cell suspensions and added to 96-well plates at a concentration of 5 × 10^4^ cells/ml, and 100 μl of cell suspension was added to each well. After a 24 h incubation with 50 MOI of Ad-VT and 4 nmol of paclitaxel, 50 MOI of Ad-VT and 4 nmol of paclitaxel mixture was added, respectively. Blank control wells were also set. After 72 h, the cells were removed and used for the WST-1 assay. The medium was discarded and 110 μl of WST-1 mixed solution (10 μl WST-1 and 100 μl DMEM medium) was added to each well and incubated in a thermostat. After a 2 h incubation, the plates were shaken for 20 s, and followed by measurement of OD values of each well at 450 nm using a microplate reader. The inhibition rate (%) of the two drugs for MCF-7, MDA-MB-231, and MCF-10A cells was calculated. Cell viability was calculated as follows: Cell inhibition ratio = 100% × (1 – absorbance of treated wells/absorbance of control wells).

### Detection of Apoptosis Levels in Breast Cancer Cells

The effects of viral and chemotherapeutic drugs on the apoptosis level of breast cancer cells were examined by Hoechst staining and Annexin V assay.

MCF-7 and MDA-MB-231 cells were prepared as cell suspensions and added to a 12-well plate at a concentration of 2 × 10^5^ cells/ml, and 1 mL of cell suspension was added to each well. After incubating for 24 h, then the cells infected with 50 MOI of Ad-VT and 4 nmol of paclitaxel, and blank control wells were also set. After 72 h, the cells were removed and stained with Hoechst dye solution. The cells were collected and washed three times, then resuspended in 100 μl PBS and stained with 1 μl of Hoechst solution. After 10 min of incubation, a 10 μl sample was applied to a microscope slide with a cover slip, and then observed and photographed under a fluorescence microscope (BX-60, Olympus, Tokyo, Japan).

MCF-7 and MDA-MB-231 cells were prepared as cell suspensions and added to a 12-well plate at a concentration of 2 × 10^5^ cells/ml, and 1 mL of cell suspension was added to each well. After incubating for 24 h, then the cells infected with 50 MOI of Ad-VT and 4 nmol of paclitaxel, and blank control wells were also set. After 72 h, the cells were removed and stained with Annexin V/FITC-PI. The cells were harvested and washed three times, resuspended in 500 μl of binding buffer, and then 5 μl of FITC solution and 5 μl of PI solution were added, followed by an incubation for 20 min in the dark. The samples were then examined by flow cytometry (FACSCalibur, Becton Dickinson, Franklin Lakes, NJ, USA) for apoptosis analysis (Cell Quest Pro, Becton Dickinson).

### Caspase Activity Analysis

MCF-7 and MDA-MB-231 cells were prepared as cell suspensions and added to a 12-well plate at a concentration of 2 × 10^5^ cells/ml, and 1 mL of cell suspension was added to each well. After incubating for 24 h, then the cells infected with 50 MOI of Ad-VT and 4 nmol of paclitaxel, and blank control wells were also set. After 72 h, the cells were collected and washed three times. The MCF-7 and MDA-MB-231 cells were resuspended with lysis buffer before total protein were extracted. Caspase-3, 6, and 7 activities were analyzed using Caspase Activity Assay Kits (Beyotime Institute of Biotechnology, Shanghai, China).

### JC-1 Staining Experiment

JC-1 can detect qualitative and quantitative changes in mitochondrial membrane potential.MCF-7 and MDA-MB-231 cells were prepared as cell suspensions and added to a 12-well plate at a concentration of 2 × 10^5^ cells/ml (cell slides were placed), and 1 mL of cell suspension was added to each well. After incubating for 24 h, then the cells infected with 50 MOI of Ad-VT and 4 nmol of paclitaxel, and blank control wells were also set. After 72 h, the cells were removed and stained with JC-1 dye solution. The medium was discarded, the wells washed three times with PBS, 1 mL of JC-1 solution was added, and the plates were incubated for 20 min in the dark. The plates were washed three times with PBS and the cells were photographed using fluorescence microscopy.

MCF-7 and MDA-MB-231 cells were prepared as cell suspensions and added to 96-well plates at a concentration of 5 × 10^4^ cells/ml, and 100 μl of cell suspension was added to each well. After incubating for 24 h, then the cells infected with 50 MOI of Ad-VT and 4 nmol of paclitaxel, and blank control wells were also set. After 72 h, the cells were removed and stained with JC-1 dye solution. After discarding the liquid, 100 μl of JC-1 solution was added to each well, and incubated for 20 min in the dark. Subsequently, the absorbances at 435 and 585 nm were measured.

### Cell Migration and Invasion Assay

MCF-7 and MDA-MB-231 cells were prepared as cell suspensions and added to a 12-well plate at a concentration of 1 × 10^5^ cells/ml, and 500 μl of cell suspension was added to each well. After incubating for 24 h, then the cells infected with 50 MOI of Ad-VT and 4 nmol of paclitaxel, and blank control wells were also set. After 72 h, the cells were harvested resuspended in 200 μl of culture medium, and then added to the upper chamber, In the lower chamber, 500 μl of culture medium containing 10% serum was added. The cells were then cultured for 24 h. Cells that migrated through the membrane were counted under a microscope after fixing them with carbinol and staining with crystal violet. The experimental procedure of the matrigel invasion assay was the same as that for the transwell migration assay; except for a 1 h incubation with matrigel (1:7 with DMEM) of the upper chamber and before seeding the cells.

### Construction and Identification of MDA-MB-231-LUC Cells

MDA-MB-231 cells were prepared as cell suspensions and added to a 6-well plate at a concentration of 1 × 10^5^ cells/ml, and 2 mL of cell suspension was added to each well. After 24 h of incubation, the cells were transfected with a mixture of 4 μg pGL4.51 plasmid (Promega, Madison, WI, USA) and 4 μl of Transfection Reagent (QIAGEN, Beijing, China). Twenty-four hours after transfection, MDA-MB-231 cells were added to a new 6-well plate at 100 cells per well. An amount of 400 μg/ml of G418 (Geneticin) (BD Bioscience Clontech, San Diego, CA, USA) was added to select the cells and the culture media was replaced every 48 h. The G418 resistant cells were passaged in 96-well plate for cell culture (withG418). When cell confluence reached 80% or more, the cells were transferred into 24-well plates and cultured. Similarly, when cell confluence reached 80% or more, they were transferred to a 12-well plate and assayed for their luciferase activity.

MDA-MB-231 cells were prepared as cell suspensions and added to 96-well plates at a concentration of 5 × 10^4^ cells/ml, and 100 μl of cell suspension was added to each well. After 48 h of incubation, the luciferase activity of each cell clone was detected using a ONE-Glo™ Luciferase Assay System (Promega, Madison, WI, USA). Subsequently, the cell clone with the highest fluorescence value was continuously cultured for 6–8 weeks, and its luciferase activity (RLU) detected every five generations, using the luciferase assay kit to ensure that the *luc* gene was stably expressed.

The characterization of MDA-MB-231-LUC cells was performed using cell growth curve and cell cycle assays. MDA-MB-231 and MDA-MB-231-LUC cells were prepared as cell suspensions and added to 96-well plates at a concentration of 5 × 10^4^ cells/ml. A 100 μl of cell suspension was added to each well and incubated for 24 h. A 96-well cell culture plate was taken at each of the seven different time points (1, 2, 3, 4, 5, 6, and 7 days), and the culture solution of each well was discarded, and 110 μl ofWST-1 solution was added, and the cells were cultured for 2 h in the dark, followed by absorbance measurement at 450 nm.

MDA-MB-231 and MDA-MB-231-LUC cells were prepared as cell suspensions and added to a 12-well plate at a concentration of 2 × 10^5^ cells/ml, and 1 mL of cell suspension was added to each well. After incubation for 24 h in an incubator, the cells were harvested and washed three times. The MDA-MB-231 and MDA-MB-231-LUC cells were resuspended in 5 mL of 75% ethanol (precooled at 4°C) and incubated at 4°C for 18 h in the dark. Subsequently, the MDA-MB-231 and MDA-MB-231-LUC cells were washed three times with PBS and then 500 μL propidium iodide (PI)/RNase solution was added. After 20 min of incubation, the sample was transferred to a labeled flow tube for flow cytometry.

### *In vivo* Analysis of the Anti-tumor Effects

The cell density of luciferase labeled human breast cancer cells was adjusted to 1 × 10^8^ cells/ml, and subcutaneously injected, in each nude mouse, with 100 μl in the right chest. After the establishment of the tumor-bearing model, the mice were randomly divided into six groups of 10 mice each and according to the tumor size: a 1 × 10^9^ PFU/100 μl Ad-VT + 20 mg/kg paclitaxel treatment group, a 1 × 10^9^ PFU/100 μl Ad-VT + 10 mg/kg paclitaxel treatment group, a 1 × 10^9^ PFU/100 μl Ad-VT treated group, a 20 mg/kg paclitaxel treated group, a 10 mg/kg paclitaxel treated group and a control group. After the subcutaneous tumor-bearing model of nude mice was successfully established, treatments with recombinant adenovirus and paclitaxel were performed by intratumoral injection every 3 days and for 3 weeks. Starting from week 0, the tumor site of the nude mice was photographed once a week, using *in vivo* living imaging equipment (Merc, Berlin, German), and photographed continuously for 6 weeks. The measurement time (week) was taken as the abscissa and the average bioluminescence value of the tumor (mean photons/s) was taken as the ordinate to plot the average bioluminescence curve of the tumor. The length and width of the xenograft tumors were measured weekly using Vernier calipers from week 0 and were continuously measured for 6 weeks. The tumor volume was calculated using the following formula: 0.52 × (smallest diameter)^2^ × (largest diameter). The percent tumor inhibition was calculated using the formula: (1 – treatment group tumor weight / control tumor weight) × 100% (26, 30, 32). After successfully establishing the xenograft models of nude mice, the survival of nude mice was recorded every day, and from 6 weeks was recorded continuously. The survival curve of the nude mice was plotted with survival time (day) as the abscissa and survival rate as the ordinate.

### Statistical Analysis

The statistical analyses were conducted using data from at least three independent experiments and using SPSS 20.0 (SPSS Inc., Chicago, IL, USA). The results were statistically analyzed by Student's *t*-tests or one-way analysis of variance (ANOVA); when one-way ANOVA results were *P* < 0.05, further multiple comparisons were performed using the Student-Newman-Keuls test. *P* < 0.05 was considered to indicate a statistically significant difference.

## Result

### Synergistic Effect Analysis

**Figure 1:**
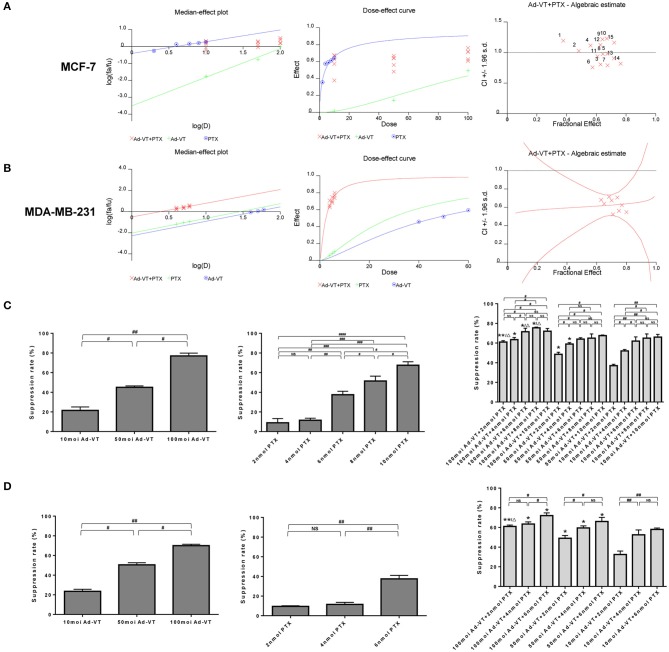
Synergistic analysis of the combination of Ad-VT and paclitaxel. **(A)** Calcusyn software analysis of the synergy between the Ad-VT and paclitaxel and evaluation of the drug concentration in MCF-7 cells. The CI of 50 MOI Ad-VT + 2 nmol paclitaxel group (CI = 0.997), 50 MOI Ad-VT + 4 nmol paclitaxel group (CI = 0.936), 50 MOI Ad-VT +6 nmol paclitaxel group (CI = 0.982), 100 MOI Ad-VT + 6 nmol paclitaxel group (CI = 0.942), 10 MOI Ad-VT + 2 nmol paclitaxel group (CI = 0.989), 10 MOI Ad-VT + 6 nmol paclitaxel group (CI = 0.975) and 10 MOI Ad-VT + 10 nmol paclitaxel group (CI = 0.977) were <1, indicating a synergistic inhibitory effect. **(B)** Synergistic effect test on MDA-MB-231 cells to determine the concentration of virus and drug. Finally, 50 MOI Ad-VT + 4 nmol (CI = 0.942) paclitaxel was selected for subsequent *in vitro* inhibition experiments. **(C)** Inhibitory effects of different concentrations of Ad-VT and paclitaxel on MCF-7 cells assessed by the WST-1 assay. **(D)** Inhibitory effects of different concentrations of Ad-VT and paclitaxel on MDA-MB-231 cells assessed by WST-1 assay. The WST-1 assays were performed in triplicate. ^#^*p* < 0.05, ^*##*^*p* < 0.01, ^*###*^*p* < 0.001, ^*####*^*p* < 0.0001. (When compared with the 10 MOI Ad-VT group at the same paclitaxel concentration, *p < 0.05, **p < 0.01; compared with the 50 MOI Ad-VT group at the same paclitaxel concentration, Δ*p* < 0.05).

The synergistic inhibitory effect of paclitaxel and Ad-VT was detected by WST-1 assay (Figures 1A–D). The purpose of this experiment was to have a synergistic effect with the combination of low-dose chemotherapeutic drugs and recombinant adenovirus, which has a higher effect of reducing toxicity and increasing efficiency. In order to select the optimal concentration, MCF-7 cells were treated with different concentrations of Ad-VT and paclitaxel. Ad-VT was used at MOI of 10, 50 and 100 and paclitaxel at 2, 4, 6, 8, and 10 nmol. By introducing the above data into the calcusyn software, we concluded that the 50 MOI Ad-VT + 2 nmol paclitaxel group, 50 MOI Ad-VT + 4 nmol paclitaxel group, 50 MOI Ad-VT +6 nmol paclitaxel group, 100 MOI Ad-VT + 6 nmol paclitaxel group, 10 MOI Ad-VT + 2 nmol paclitaxel group, 10 MOI Ad-VT + 6 nmol paclitaxel group, and 10 MOI Ad-VT + 10 nmol paclitaxel group were <1, indicating a synergistic inhibitory effect. The CI of the other combinations was >1, indicating antagonistic effect. Based on the above results, we further narrowed the concentration range of paclitaxel to 2, 4, and 6 nmol.

Subsequently, we performed a synergistic effect test on MDA-MB-231 cells to further determine the concentration of virus and drug. After using the concentration that is summarized above, in MCF-7 and MDA-MB-231 cells, 50 MOI Ad-VT + 4 nmol paclitaxel was selected for subsequent *in vitro* inhibition experiments (Figures 1C,D). At this concentration, the combination of Ad-VT and paclitaxel had a good synergistic effect, and the inhibition rate of paclitaxel on tumor cells was ~10%, and the inhibition rate of Ad-VT on tumor cells was ~40%. In this case, the combination of drugs reflects the effect of synergy and the effect of reducing toxicity.

### The Combination of Ad-VT and Paclitaxel Can Increase the Inhibition of Breast Cancer Cells and Reduce Drug Toxicity

In order to analyze whether the combination of Ad-VT and paclitaxel could reduce toxicity and enhance efficacy, we performed crystal violet staining and WST-1 experiments using breast cancer cells and normal breast epithelial cells.

**Figure 2:**
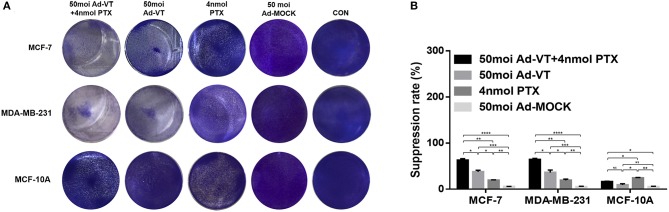
Inhibitory effect of the combination Ad-VT and paclitaxel on MCF-7, MDA-MB-231, and MCF-10A cells. **(A)** MCF-7, MDA-MB-231, and MCF-10A cells were infected with Ad-VT, Ad-MOCK, paclitaxel, and Ad-VT and paclitaxel mixture, then stained with 0.4% Crystal Violet at 72 h. Ad-VT, paclitaxel and the combination of Ad-VT and paclitaxel exerted inhibitory effects on MCF-7 and MDA-MB-231 cells. Ad-VT is substantially non-toxic to MCF-10A cells. Paclitaxel showed a cytotoxic effect in MCF-10A cells, and this was reduced when Ad-VT and paclitaxel were combined. **(B)** MCF-7, MDA-MB-231, and MCF-10A cells viability were determined using WST-1 assays after infection with Ad-VT, paclitaxel and Ad-VT and paclitaxel mixture for 72 h. The inhibitory effect of combination Ad-VT and paclitaxel group had a significantly difference with other groups on breast cancer cells. All measurements were performed in triplicate. *p < 0.05, **p < 0.01, ***p < 0.001, ****p < 0.001.

The crystal violet staining results showed that Ad-VT can cause obvious cytotoxicity in MCF-7 and MDA-MB-231 cells, but was substantially non-toxic to MCF-10A cells (Figure 2A). Paclitaxel showed a certain cytotoxic effect in MCF-10A cells, with reduced toxicity when Ad-VT and paclitaxel were combined (Figure 2A).

In the WST-1 experimental results, we also found that Ad-VT has significant inhibitory effect on both breast cancer cells (Figure 2B). The inhibition rate could reach ~40%, and had no toxic effect on normal breast epithelial cells, while paclitaxel had significant cytotoxicity on normal breast epithelial cells (*P* < 0.01). This effect was significantly reduced when Ad-VT and paclitaxel were combined (*P* < 0.05). The inhibitory effect on breast cancer cells, after combination therapy, had an inhibitory rate that was higher than 65% and was also significantly higher than that of Ad-VT or paclitaxel alone (*P* < 0.05). The above results indicate that the combination of Ad-VT and paclitaxel can significantly increase the inhibitory effect on breast cancer cells and reduce the toxicity of paclitaxel.

### Combination of Ad-VT and Paclitaxel Can Increase Apoptosis in Breast Cancer Cells

**Figure 3:**
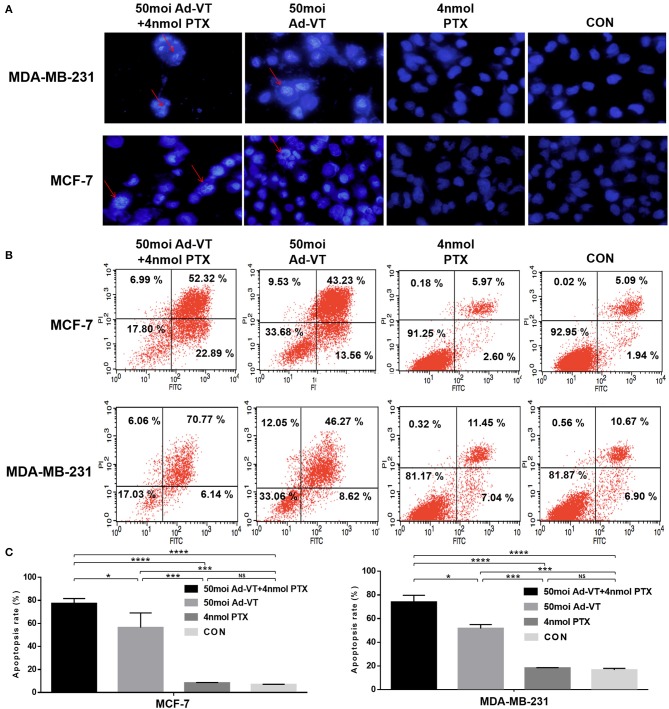
Identification of MCF-7 and MDA-MB-231 cells apoptosis induced by the combination Ad-VT and paclitaxel using Hoechst staining and Annexin V assays. **(A)** Morphological changes visualized by fluorescence microscopy after Hoechst staining. MCF-7 and MDA-MB-231 cells were infected with Ad-VT, paclitaxel, and Ad-VT and paclitaxel mixture, stained with Hoechst stain at 72 h. Nuclear thickening and nuclear fragmentation increased significantly with time in Ad-VT and in the combination Ad-VT and paclitaxel groups. **(B)** MCF-7 and MDA-MB-231 cells apoptosis was analyzed by flow cytometry after Annexin-V FITC/PI staining. MCF-7 and MDA-MB-231 cells infected with Ad-VT and combination of Ad-VT and paclitaxel exhibit abundant apoptosis. All measurements were performed in triplicate. *p < 0.05, ***p < 0.001, ****p < 0.0001.

In order to verify whether the inhibition of cancer cells growth derived from traditional apoptotic pathways, Hoechst staining was further performed. The Hoechst staining results showed that Ad-VT could increase the apoptosis of MCF-7 and MDA-MB-231 cells, as determined by the presence of cells with nuclear bright blue hyperchromatism and nuclear fragmentation (Figure 3A). This event was not observed in the paclitaxel treated group. After the combination of Ad-VT and paclitaxel, the proportion of apoptotic cells, was higher than that in the Ad-VT group.

After qualitative analysis by Hoechst staining, we further analyzed the apoptosis of breast cancer cells by flow cytometry. From analyzing the Annexin V flow cytometry results, we also found that the apoptosis level of paclitaxel treatment was not significantly different from that of the control group, while the apoptosis level of Ad-VT group was ~55% significantly higher than that of the control group (Figures 3B,C). After the combination of Ad-VT and paclitaxel, the apoptosis level of the two types of breast cancer cells was significantly increased and reaching ~75% (*p* < 0.05). The above results indicate that Paclitaxel combined with Ad-VT can synergistically increase apoptosis of breast cancer cells.

### Combination of Ad-VT and Paclitaxel Induces Apoptosis Through the Mitochondrial Pathway

**Figure 4:**
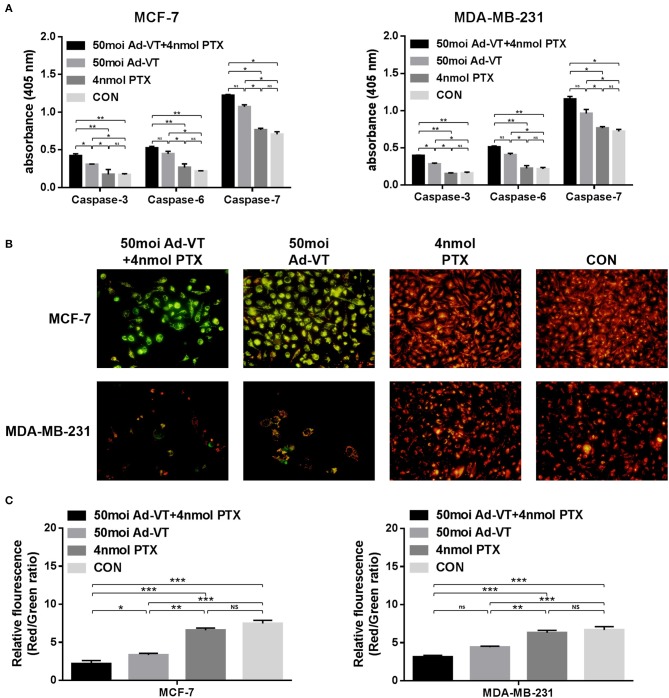
Analysis of mitochondrial membrane potential by JC-1 staining and analysis of caspase activation in MCF-7 and MDA-MB-231 cells infected with a combination Ad-VT and paclitaxel. **(A)** Cells were treated with the Ad-VT, paclitaxel and Ad-VT and paclitaxel mixture for 72 h. The vertical coordinate for the content of caspases was in units of μg/μl. The levels of caspases-3, 6, and 7 increased significantly in Ad-VT and in the combination Ad-VT and paclitaxel groups. **(B)** Changes in red and green fluorescence measured by fluorescence microscopy after JC-1 staining. Increased apoptosis results in a decrease in the ratio of red to green fluorescence. **(C)** Quantitative measurement of changes in the ratio of red to green fluorescence after JC-1 staining. Ad-VT and the combination Ad-VT and paclitaxel clearly altered the mitochondrial membrane potential, and combination of Ad-VT and paclitaxel had the strongest ability to induce apoptosis by affecting the mitochondrial membrane potential. All measurements were performed in triplicate. *p < 0.05, **p < 0.01, ***p < 0.001.

As a result of the Caspase activity assay, Ad-VT significantly increased the levels of caspases-3, 6, and 7 in MCF-7 and MDA-MB-231 cells, and their levels did not significantly change in paclitaxel-treated cells. After the combination, the levels of caspases-3, 6, and 7 in the treated cells, were significantly higher than those in the Ad-VT group (Figure 4A).

In order to verify whether the combination of Ad-VT and paclitaxel inhibits the growth of breast cancer cells by the intrinsic apoptotic pathway, we performed JC-1 staining *in vitro*. The JC-1 staining showed that Ad-VT obviously affected the changes of mitochondrial membrane potential (MMP) (Figure 4B). Ad-VT caused a severe damage to mitochondria, while paclitaxel-treated cells showed no obvious changes in mitochondrial membrane potential. The combination of Ad-VT and paclitaxel induced obvious changes in the mitochondrial membrane potential of the two breast cancer cell lines. In the red-green ratio results, we also found that the mitochondrial membrane potential of the Ad-VT group was significantly higher than that of the control group and that of the paclitaxel-treated group (*p* < 0.01) (Figure 4C). This effect was more significant with the combination treatment. These results indicate that the combination of Ad-VT and paclitaxel could induce apoptosis of breast cancer cells by activating the endogenous apoptotic pathway.

### The Combination of Ad-VT and Paclitaxel Can Increase the Inhibition of Breast Cancer Cell Migration and Invasion

**Figure 5:**
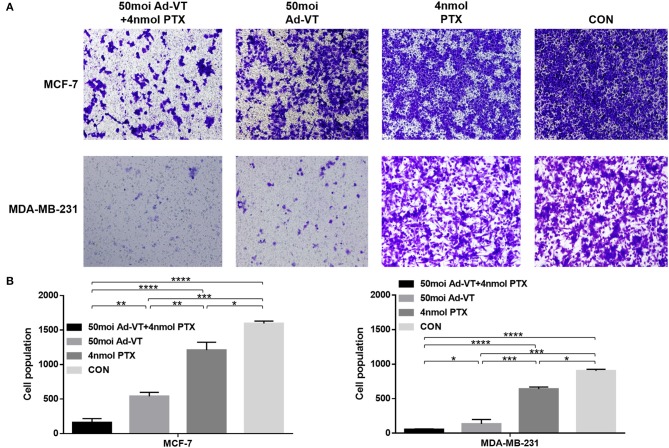
Effects on MCF-7 and MDA-MB-231 cells migration assessed using the Transwell assay. **(A)** Suppression of MCF-7 and MDA-MB-231 cells migration 72 h after treatment with Ad-VT, paclitaxel, and Ad-VT and paclitaxel mixture. **(B)** Cells that had migrated through the membrane were counted under a microscope after fixation with carbinol and staining with crystal violet. Cells infected with the combination Ad-VT and paclitaxel showed the lowest migration at 72 h. All measurements were performed in triplicate. *p < 0.05, **p < 0.01, ***p < 0.001, ****p < 0.0001.

**Figure 6:**
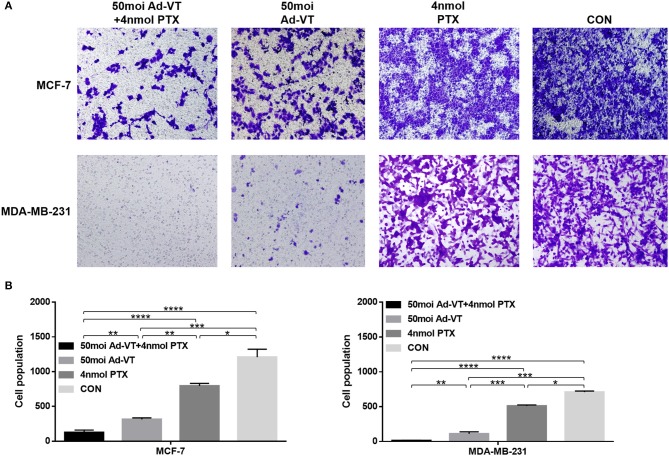
Effects on MCF-7 and MDA-MB-231 cells invasion assessed using the Transwell assay. **(A)** Suppression of MCF-7 and MDA-MB-231 cells invasion 72 h after treatment with Ad-VT, paclitaxel and Ad-VT and paclitaxel mixture. **(B)** Cells that passed through the membrane were counted under a microscope after fixation with carbinol and staining with crystal violet. Cells infected with the combination of Ad-VT and paclitaxel, showed the lowest invasion at 72 h. All measurements were performed in triplicate. *p < 0.05, **p < 0.01, ***p < 0.001, ****p < 0.0001.

We also analyzed whether the combination of Ad-VT and paclitaxel could increase the ability of the drugs to inhibit breast cancer cells migration and invasion. In the transwell invasion assay, we found that the combination treatment with Ad-VT and paclitaxel inhibits the migration ability of MCF-7 and MDA-MB-231 cells (Figures 5A,B). The effect of Ad-VT was significantly higher than that of paclitaxel, and the effect after combination therapy was significantly higher than that of the Ad-VT group and the paclitaxel group (*p* < 0.01). Similar results were observed in the Transwell invasion assay (Figures 6A,B). The above results indicate that the combination of Ad-VT and paclitaxel significantly increases the inhibitory effect on migration and invasion of breast cancer cells.

### Construction and Identification of MDA-MB-231-LUC

**Figure 7:**
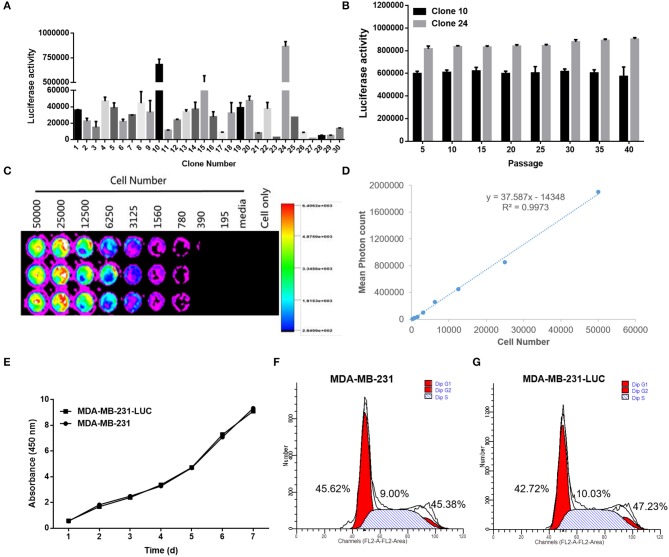
Screening and identification of MDA-MB-231-LUC cells. **(A)** After transfection with the pGL4.51 plasmid, the two cell clones with the highest luciferase activity, were screened with 400 μg/ml of G418. **(B)** The luciferase activity of each cell clone was detected using a ONE-Glo™ Luciferase Assay System. The luciferase activity (RLU) of cell clones were detected every five generations using the luciferase assay kit to determine whether the *Luc* gene was stably expressed. **(C,D)** The cell clone with the highest luciferase activity was inoculated in a 1:2 ratio into a 96-well plate. After adding fluoresce in, the relationship between bioluminescence intensity and cell number was observed. The cell bioluminescence intensity increased with the increase of cell number and Clone 24 was found to stably express luciferase. **(E)** The MDA-MB-231 and MDA-MB-231-LUC cells were cultured in 96-well cell culture plates, and cell growth trends were detected at 1, 2, 3, 4, 5, 6, and 7 days. **(F,G)** The MDA-MB-231 and MDA-MB-231-LUC cells were cultured in 12-well cell culture plates, and cell cycles were detected using flow cytometry.

After detecting the fluorescence activity (RLU) of each cell clone, we found that clone 10 and clone 24 had the highest fluorescence activity. Clone 10 and clone 24 were cultured for multiple generations, and after each five generations of fluorescence activity detection, we found that both cells stably expressed the fluorescence in protein. The fluorescent activity of clone 24 was higher than that of clone 10 (Figures 7A,B). Therefore, clone 24 was selected for subsequent experiments.

Clone 24 was sequentially diluted and seeded into a 96-well plate at a ratio of 1:2. After the addition of the fluoresce in substrate, the bioluminescence intensity and cell number of MDA-MB-231-LUC presented a certain linear relationship (*R*^2^ = 0.9973). The luminescence intensity increased with the increase in cell number, further proving that Clone 24 stably express luciferase (Figures 7C,D).

The biological characteristics of MDA-MB-231 and MDA-MB-231-LUC cells were subsequently compared. The results showed that the growth trends of the two cell lines were similar, and no significant differences were found in the cell cycle test results (Figures 7E–G). The above results indicate that the stable expression of *Luc* did not affect the growth characteristics of the cells (*P* > 0.05).

### The Combination of Ad-VT and Paclitaxel Inhibits Tumor Growth and Reduces Toxicity *in vivo*

**Figure 8:**
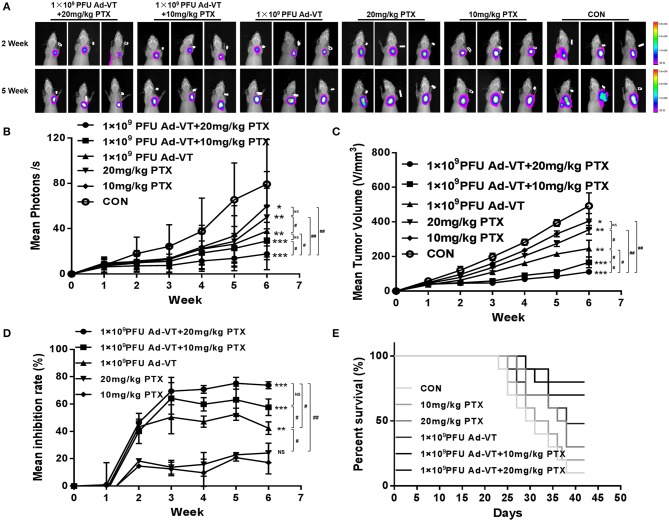
Effect of recombinant adenoviruses on breast cancer in a BALB/c nude mouse model. **(A,B)** The xenograft models were established *via* subcutaneous injection of MDA-MB-231-LUC cells (1 × 10^7^/100 μl) into the right axil of the mice (*n* = 10 per group). *In vivo* bioluminescent imaging was used to continuously monitor changes in tumor bioluminescence intensity. **(C,D)** Length and width of xenograft tumors measured weekly for 6 weeks using Vernier calipers. The average tumor inhibition was calculated using the formula: (1 – treatment group tumor weight / control tumor weight) × 100%. **(E)** After successfully establishing xenograft models in nude mice, the mice survivals were recorded every day for 6 weeks. The average tumor inhibition of the 1 × 10^9^ PFU/100 μl Ad-VT + the 20 mg/kg paclitaxel treated group was significantly higher than that of the other groups and reaching 73.1%. Similarly, the survival rate of the 1 × 10^9^ PFU/100 μl Ad-VT + the 20 mg/kg paclitaxel treated group was also the highest, reaching 80%. ^#^*p* < 0.05, ^*##*^*p* < 0.01. (*p < 0.05, **p < 0.01, ***p < 0.001 vs. Control or 10 mg/kg PTX treated group).

After analyzing the inhibitory effect *in vitro*, we tested the tumor inhibitory effect in a nude mice subcutaneous tumor-bearing model. The change in the bioluminescence intensity of the tumor was observed using a living body imaging system that was continuously monitored for 6 weeks (Figures 8A,B). From the 2nd week, the tumor bioluminescence intensity of the control group, began to increase rapidly, and from the third week, it was higher than that of the other groups. At 2–3 weeks, and in addition to the control group, the average bioluminescence intensity of the tumors in the other treated groups, increased slightly. At 3–6 weeks, the average bioluminescence intensity of the 1 × 10^9^ PFU/100 μl Ad-VT + 20 mg/kg paclitaxel treated group, was always lower than that of the other treated groups. At 6 weeks, the average bioluminescence intensity of the 1 × 10^9^ PFU/100 μl Ad -VT + 20 mg/kg paclitaxel treated group and the 1 × 10^9^ PFU/100 μl Ad-VT + 10 mg/kg paclitaxel treated group, was significantly lower than that of the control group (*P* < 0.05). The results of tumor volume measurement were similar to the mean luminescence intensity (Figure 8C). The inhibitory effect of Ad-VT combined with paclitaxel was significantly higher than that of the virus or drug alone.

From the tumor growth inhibition curve, it could be seen that the average tumor inhibition rate of the 20 mg/kg paclitaxel treated group and the 10 mg/kg paclitaxel treated group, was significantly lower than in the other treated groups (*P* > 0.05) (Figure 8D). At 2–6 weeks, the average tumor inhibition rate of the 1 × 10^9^ PFU/100 μl Ad-VT + the 20 mg/kg paclitaxel treated group, was always higher than that of the other groups. At 6 weeks, the average tumor inhibition rate of the 1 × 10^9^ PFU/100 μl Ad-VT + the 20 mg/kg paclitaxel treated group, was significantly higher than that of the other treated groups and the control group (*P* < 0.05). The average tumor inhibition rate of the 1 × 10^9^ PFU/100 μl Ad-VT + the 20 mg/kg paclitaxel treated group reached 73.1% at the highest, indicating that the combination of Ad-VT and paclitaxel significantly inhibits tumor growth *in vivo*.

The mouse survival rate results showed that compared to the other treated groups, the mice of the control group began to die from ~23 days after the formation of the subcutaneous tumors, and the average survival times were about 31.8 days (Figure 8E). The mice of the 20 mg/kg paclitaxel treated group and the 10 mg/kg paclitaxel treated group began to die ~25 days after the formation of the subcutaneous tumors, and the average survival times were ~33.2 and 33.6 days, respectively. Compared with the control group, the 20 mg/kg paclitaxel treated group and the 10 mg/kg paclitaxel treated group, the average survival time of the Ad-VT treated groups was ~37.4 days (*P* < 0.05). After the combination, the survival of the mice was significantly prolonged, the average survival times of the 1 × 10^9^ PFU/100 μl Ad-VT + the 20 mg/kg paclitaxel treated group and the 1 × 10^9^ PFU/100 μl Ad-VT + 10 mg/kg paclitaxel treated group, were ~40.2 and 39.6 days, respectively. At 42 days, the survival rate of the two treated mice were 80 and 70%, respectively. This indicates that the combination of Ad-VT and paclitaxel can increase the survival rate of mice and significantly prolong their survival.

## Discussion

Cancer incidence and mortality are rapidly growing worldwide. It is the second leading cause of death in developing countries, and it is estimated that in 2018, there will be 18.1 million new cases and 9.6 million cancer deaths worldwide (33–35). Breast cancer is the most common malignant tumor in women worldwide. The number of patients is significant and continues to increase, which seriously threatens women's health and quality of life. The molecular mechanisms of breast cancer pathogenesis is unclear, due to the involvement of various pathogenic factors. The treatment of breast cancer patients involves surgery, radiation, chemical, endocrine therapy, and HER2 molecular targeted therapy.

For patients with early breast cancer, surgery and chemotherapy are the main treatments. However, after chemotherapy, there are often adverse reactions, such as allergic reactions, myelosuppression, neurotoxicity, cardiovascular toxicity, gastrointestinal reactions, and liver toxicity. Several reports showed that the 5-years survival rate of patients is 26% (34). Although imaging screening has significantly improved the detection rate of early breast cancer, late detection of the disease in some patients with recurrence and metastasis results in poor treatment. There is currently no standard treatment for advanced breast cancer and attempts using multiline therapy were unsuccessful.

In recent years, with the development of biomedical and genetic engineering technologies, many new therapeutic programs have been developed, including gene-targeted and oncolytic virotherapy (36).

Oncolytic viruses are a type of replicative tumor-killing viruses that selectively infect and replicate in tumor cells, having the effect of killing tumor. Compared with other types of tumor therapies, oncolytic viruses have the following advantages: (1) They have a multi-path killing mechanism, with a broad anti-tumor spectrum, that is effective against recurring and metastatic tumors; (2) They are safe and reliable, have lower toxic side effects, and are beneficial when combined with other immunotherapy and anti-cancerous drugs; (3) Compared with cell immunotherapy such as CAR-T, the production and the treatment costs are low.

Due to their outstanding performance, especially in the development and clinical use of adenoviral vector drugs, adenoviruses have attracted significant attention in the field of oncolytic virotherapy. Therefore, the strategy of targeting tumors with an adenovirus as a vector, is expected to replace traditional cancer therapy. Simultaneously, and in previous studies, we took advantage of the characteristics of apoptin and the *hTERT* promoter, to construct a tumor-specific replicative recombinant adenovirus that expresses apoptin (Ad-Apoptin-hTERTp-E1a, Ad-VT) (26). This allowed the adenovirus to specifically replicate in tumor cells, proliferate in large numbers, and express the apoptin protein, which leads to tumors cell death.

Apoptin is a protein that specifically induces apoptosis in tumor cells without affecting most of normal cells. Since apoptin function is not mediated by p53 and is not inhibited by Bcl-2 over-expression, it is considered a novel anti-tumor protein. The *hTERTp* promoter is a tumor-specific promoter that activates the replication and/or expression of certain genes in tumor cells, such as the adenoviral promoter *E1a* gene and the gene encoding Apoptin; thereby, conferring specific replication and killing abilities to recombinant adenovirus. The presence of these two proteins (apoptin and hTERTp) can effectively enhance the tumor targeting effect of oncolytic adenovirus, and hence improve the anti-tumor effects of treatments.

In 2015, the FDA approved T-VEC (Talimogenelaherparepvec, Imlygic), a recombinant herpes simplex virus product carrying the human granulocyte-macrophage colony-stimulating factor (GM-CSF). In 2016, T-VEC was approved for marketing in Europe and Canada; thus, marking the maturity of oncolytic virus technology and its formal recognition for cancer treatment. At present, the sales of oncolytic virus products that are approved for marketing in various countries, are flat and the route of administration is limited to intratumoral injection; the main reasons are the limited response rate of monotherapy, and the approved indications are mainly for melanoma tumors (37–40). Therefore, most researchers turned their attention to a combined treatment approach.

Studies have shown that the combination therapy of Keytruda PD-1 antibody with Imlygic oncolytic virotherapy (T-VEC) in patients with melanoma, can increase patients' response rate to 62%, which is much higher than the expected remission rates of Keytruda or T-VEC alone (~35–40%) (41). In addition, studies have also shown that the overall response rate of melanoma patients treated with a combination of the oncolytic virus Imlygic (T-VEC) and the checkpoint inhibitor CTLA-4 antibody, has doubled compared with a single treatment with Yervoy (Ipilimumab) CTLA-4 antibody (42). These results show that oncolytic virotherapy combined with other tumor treatment methods can obtain significant therapeutic effects.

In the present study, we analyzed the synergistic concentration of Ad-VT and the chemotherapeutic drug paclitaxel and studied the inhibitory effect of combining Ad-VT and paclitaxel on breast cancer cells. In MCF-7 and MDA-MB-231 cells, Ad-VT and paclitaxel inhibited breast cancer cells, and the inhibition rate was 40 and 10%, respectively. After a combined application, the inhibition rate of both breast cancer cells reached more than 65%, indicating that the combination of Ad-VT and paclitaxel has significant synergistic effects. In normal mammary epithelial cells, Ad-VT has no significant toxic effects on cells, and paclitaxel has significant toxicity. Cell toxicity following the combination of Ad-VT and paclitaxel was significantly reduced, indicating that the combination of treatment can not only improve the tumor inhibition effect, but also reduces the toxicity of chemotherapy drugs on normal cells.

Studies have shown that the inoculation of Ad-shVEGF with adenovirus as a vector, in tumor-bearing mice, can induce strong inhibitory effects on tumor anti-angiogenesis (43). Another recent study showed that micro RNAs (miRNAs) based on the AdC 68 vector can down-regulate the high expression of survivin in tumors, and causes mitotic and cell cycle arrests at the G2/M phase (44). In nude mice model of tumor xenografts, miRNAs targeting survivin expressed by rAdC 68, effectively delayed the growth of liver and cervical cancers (44). In the present study, recombinant viruses Ad-VT constructed using an adenovirus, were also used to inhibit MCF-7 and MDA-MB-231 cells, and with significant inhibitory effects.

In this study, the plasmid pGL4.51 was used to transfect and generate tumor cells that stably express luciferase (MDA-MB-231-LUC) and that can allow to visualize tumor growth in our *in vivo* model. Throughout the experiment, we found that the average tumor luminescence intensity of the control group increased significantly and was significantly higher than in the other treated groups. At 3–6 weeks, the average bioluminescence intensity of the 1 × 10^9^ PFU/100 μl Ad-VT + the 20 mg/kg paclitaxel treated group was lower than that of the other treated groups. At 4–6 weeks, the average bioluminescence intensity of the 1 × 10^9^ PFU/100 μl Ad -VT + the 20 mg/kg paclitaxel treated group and the 1 × 10^9^ PFU/100 μl Ad-VT + the 10 mg/kg paclitaxel treated group was significantly lower than that of the control group (*P* < 0.05). From the results of the tumor inhibition rate, we also found that the average tumor inhibition rate of the 1 × 10^9^ PFU/100 μl Ad-VT + the 20 mg/kg paclitaxel treatment group was always higher than that of the other groups. At 5 weeks, the average tumor inhibition rate of the Ad-VT treated group was as high as 73.1%. In the mouse survival rate results, we also found that after the combination, the survival of the mice was significantly prolonged, and the average survival times of the 1 × 10^9^ PFU/100 μl Ad-VT + the 20 mg/kg paclitaxel treated group and the 1 × 10^9^ PFU/100 μl Ad-VT + the 10 mg/kg paclitaxel treated group, were ~40.2 and 39.6 days, respectively. At 42 days, the survival rate of the two treated mice were 80 and 70%, respectively. These results indicate that the combination of Ad-VT and paclitaxel can significantly inhibit tumor growth *in vivo* and increase the survival rate of mice.

The inhibition pathway in treated breast cancer cells with the combination of Ad-VT and paclitaxel was analyzed using caspase activity analysis, JC-1 staining, Hoechst staining and Annexin V-FITC/PI flow assays. The results showed that after the treatment combination, the apoptosis level of the two types of breast cancer cells was significantly increased, reaching ~75%, and the mitochondrial membrane potential of the Ad-VT group was significantly higher than that of the control group (*p* < 0.01). This effect was more significant with the treatment combination. These results indicate that the combination of Ad-VT and paclitaxel can induce apoptosis of breast cancer cells by activating the endogenous apoptotic pathway.

In conclusion, the recombinant adenovirus Ad-VT has a significant inhibitory effect on breast cancer cells, and without having a toxic effect on normal breast epithelial cells; while, paclitaxel has a cytotoxic effect on normal cells. After combining Ad-VT and paclitaxel, we found that the synergistic treatment inhibited breast cancer cells, with significantly reduced toxicity in normal breast epithelial cells. The findings provide a theoretical basis for the treatment of breast cancer using oncolytic adenovirus combined with chemotherapy.

## Data Availability Statement

The data that support the findings of this study are available from the corresponding author upon reasonable request.

## Ethics Statement

The animal study was reviewed and approved by Institutional Animal Care and Use Committee (IACUC) of the Chinese Academy of Military Medical Science (10ZDGG007).

## Author Contributions

JW, XLi, LS, and NJ conceived and designed the experiments. JW, YL, SL, WY, XLiu, YZ, WL, and XL performed the experiments. JW, XLi, LS, and NJ analyzed the data. YL, SL, WY, XLiu, YZ, and WL contributed reagents, materials and analysis tools. JW and XLi wrote the manuscript. All authors read and approved the final manuscript.

### Conflict of Interest

The authors declare that the research was conducted in the absence of any commercial or financial relationships that could be construed as a potential conflict of interest.
